# Defining the dystrophic femoral neck in osteogenesis imperfecta: a radiographic and anatomical entity with diagnostic threshold

**DOI:** 10.1051/sicotj/2026001

**Published:** 2026-07-03

**Authors:** Samuel Georges, Jad Zalaket, Alina Badina, Stephanie Pannier, Ibrahim Saliba, Zagorka Pejin

**Affiliations:** Service de Chirurgie orthopédique et traumatologie pédiatrique, Hôpital Necker Enfants malades - AP-HP 149 Rue de Sèvres 75015 Paris France; 2 Genetics Department, Paris Cité University, INSERM UMR 1163, Imagine Institutee 75015 Paris France

**Keywords:** Osteogenesis imperfecta, Femoral neck, Hip anatomy, Acetabular protrusion, Bone dysplasia

## Abstract

*Introduction*: Severe Osteogenesis Imperfecta (OI) can cause distinct proximal femoral deformities, but specific femoral neck changes remain poorly defined. This study aimed to characterize dystrophic femoral neck morphology and determine a diagnostic threshold. *Methods*: We retrospectively reviewed anteroposterior pelvic radiographs from patients >8 years old with severe OI (*n* = 24 hips) and age-matched controls (*n* = 24 hips). Measurements included femoral neck length, neck diameter, head diameter, neck-shaft angle, anterior lateral proximal femoral angle (aLPFA), and acetabular protrusion. Ratios of neck length-to-diameter and neck length-to-head diameter were calculated. Statistical comparisons used t-tests; ROC analysis identified the optimal threshold for distinguishing dystrophic necks. *Results*: OI patients had shorter femoral necks (52.7 ± 9.9 mm vs. 64.8 ± 9.6 mm, *p* < 0.001), smaller diameters (19.4 ± 4.7 mm vs. 32.8 ± 4.8 mm, *p* < 0.0001), and higher neck length-to-diameter ratios (2.82 ± 0.63 vs. 1.98 ± 0.19, *p* < 0.000001). A threshold of ≥2.35 (AUC = 0.90) identified dystrophic necks, associated with greater acetabular protrusion (–2.91 ± 12.33 mm vs. 4.42 ± 8.67 mm, *p* = 0.004). *Discussion*: A neck length-to-diameter ratio ≥ 2.35 reliably defines dystrophic femoral necks in OI and correlates with increased acetabular protrusion. Early recognition may guide surgical planning and help preserve hip function. *Level of Evidence*: Level IV – Retrospective comparative study.

## Introduction

Lower limb deformities in patients with Osteogenesis Imperfecta (OI) are closely associated with disease severity [1, 2]. Recurrent femoral neck and diaphyseal fractures, as well as overall bone fragility, are influenced both by the severity of the disease and by factors such as the absence of weight-bearing, hip immobility, and reduced physical activity [3]. These factors contribute to deformities that alter the anatomical and mechanical axes of the femur in all three planes: coronal, sagittal, and axial. Varus deformity of the femoral neck, along with cortical thinning, adversely affects hip biomechanics and may play a critical role in the progression of deformities, increased fracture risk, and overall morbidity [4, 5].

These deformities may also directly affect the acetabulum, pelvic alignment, and spinal posture, leading to reduced hip mobility and impaired quality of life. Despite their clinical importance, these specific anatomical changes remain poorly characterized in the literature [6, 7].

When overlooked in the initial assessment by orthopedic surgeons, these femoral abnormalities can negatively impact surgical planning and lead to suboptimal outcomes [8–12]. Conversely, accurate identification and diagnosis may inform treatment strategies, improve outcomes, enhance quality of life, and reduce the need for multiple surgical interventions.

This study aims to characterize and classify the anatomical and radiological features of dystrophic femoral necks in patients with OI. We hypothesize that elongated and thinned femoral necks, often associated with reduced neck-shaft angles, represent a distinct dystrophic pattern in this population.

## Methods

We conducted a retrospective review at our institution, a reference center for pediatric and rare diseases, covering the period from 2000 to 2025. The study received approval from the institutional review board. Medical records and imaging data were reviewed for patients aged over 8 years who had anteroposterior (AP) pelvic radiographs available. Hips from patients with severe Osteogenesis Imperfecta (OI) were compared to those from age-matched controls without known bone pathology. Demographic data were collected, and all radiographic measurements were performed by a single observer. The Sillence classification and results from genetic testing were recorded for patients with OI.

**Figure 1:**
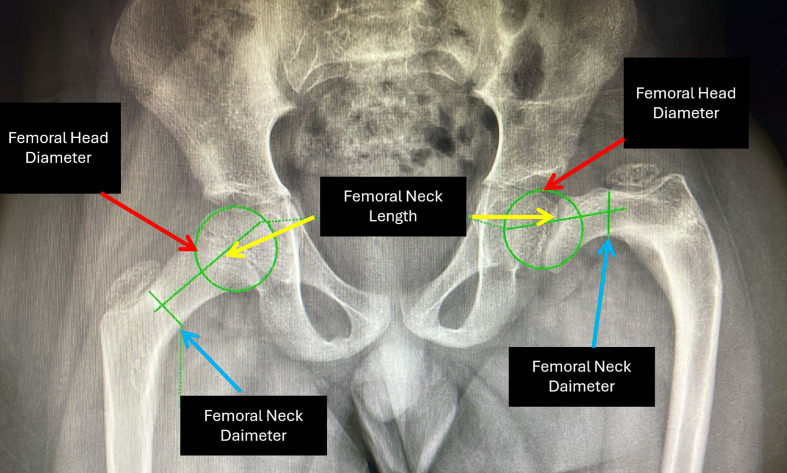
Femoral neck measurements on an anteroposterior (AP) pelvic radiograph.

Radiographic analysis included measurements of the neck-shaft angle (degrees), femoral neck length (cm), and femoral neck diameter, defined as the narrowest point of the femoral neck (cm). The diameter of the femoral head was also measured (Figure 1). Two ratios were calculated: femoral neck length to neck diameter, and femoral neck length to femoral head diameter. Proximal femoral morphology was further evaluated using the anterior Lateral Proximal Femoral Angle (aLPFA). Acetabular involvement was assessed by measuring the distance between the acetabular floor and the ilio-ischial line (mm), and protrusio acetabuli was graded according to the Sotelo-Garza and Charnley classification [13].

Statistical comparisons between the OI and control groups were performed using unpaired Student’s *t*-tests. Pearson’s correlation coefficient was used to evaluate the relationship between femoral neck morphology and acetabular protrusion within the OI group. Receiver operating characteristic (ROC) curve analysis was conducted to identify an optimal cut-off value for any ratio that showed a statistically significant difference between groups, to distinguish dystrophic femoral necks. A *p*-value less than 0.05 was considered statistically significant.

## Results

A total of 48 hips from 48 patients were analyzed. The overall mean age was 15.7 ± 10.0 years, with a female-to-male ratio of 1.7:3. In the OI group, we identified three patients with Sillence type 4, one patient with type 5, one patient with type 1, and nineteen patients with type 3, including six cases associated with Bruck syndrome. The specific gene mutations were also recorded when available (Table 1).

**Table 1:** Demographics of patients with osteogenesis imperfecta. Type of Sillence classification and gene mutation are noted. M: male. F: Female. Sd: syndrome.

Patient	Sex	Age	Type OI	Gene mutation
1	M	16	4	c.2314G>A (p.(Gly772ser)) heterozygous exon 38 gene **COL1A2**
2	M	11	3	**COL1A2** c.2314G>A p.Gly772Ser
3	F	9	4	**SERPINF1** c.413G>A o.Ser 138 Asn
4	M	15	3	c.3106G>C (p.Gly1036Arg) heterozygous exon 47 gene **COL1A2**
5	M	9	3+Bruck sd	**FKBP10** NM_021939.3 c.831dup Exon 5 .Gly278Argfs*95
6	M	9	3+Bruck sd	**type FKPB10**
7	M	10	3	**COL1A2** NM_00008 c.2026-1_2042dup Exon 34 p.Ala680_Gly685dup
8	M	10	3	**COL1A2**
9	M	10	3	De novo gene mutation **COL1A1**
10	M	10	3	**COL1A1**
11	F	14	1	variant **COL1A1**
12	F	8	3+Bruck sd	**FKBP10** NM_021939.3 c.1276C>T p.Gln426*
13	F	8	3+Bruck sd	type FKPB10
14	M	22	3+Bruck sd	**type FKPB10** forme récessive
15	M	22	3+Bruck sd	**type FKPB10**
16	F	12	3	NA
17	F	17	3	NA
18	F	17	3	NA
19	M	19	3	**COL1A12**
20	M	17	3	NA
21	M	17	3	NA
22	F	58	4	**COL1A2** NM_000089.3 c2324G exon 38 P.Gly775 Glu p.Ala680_Gly685dup
23	M	21	3	mutation c.2882G>A hétérozygote exon 44 du gène **COL1A2**
24	M	36	5	variant c.-14C>TA (p.?) heterozygous simple exon 1 gene **IFITM5**

Among these, 24 hips were from patients with severe Osteogenesis Imperfecta (OI) and 24 from age-matched controls. The mean age in the OI group was 16.1 ± 10.6 years, and 15.3 ± 9.4 years in the control group, with no significant difference (*p* = 0.76). The female-to-male ratio was 1:2 in the OI group (8 females, 16 males) and 3:5 in the control group (9 females, 15 males), also without a significant difference (*p* = 0.76). Right hips were more commonly analyzed in both groups (13 right, 11 left in the OI group; 17 right, 7 left in controls), but side distribution was not significantly different (*p* = 0.22).

**Figure 2:**
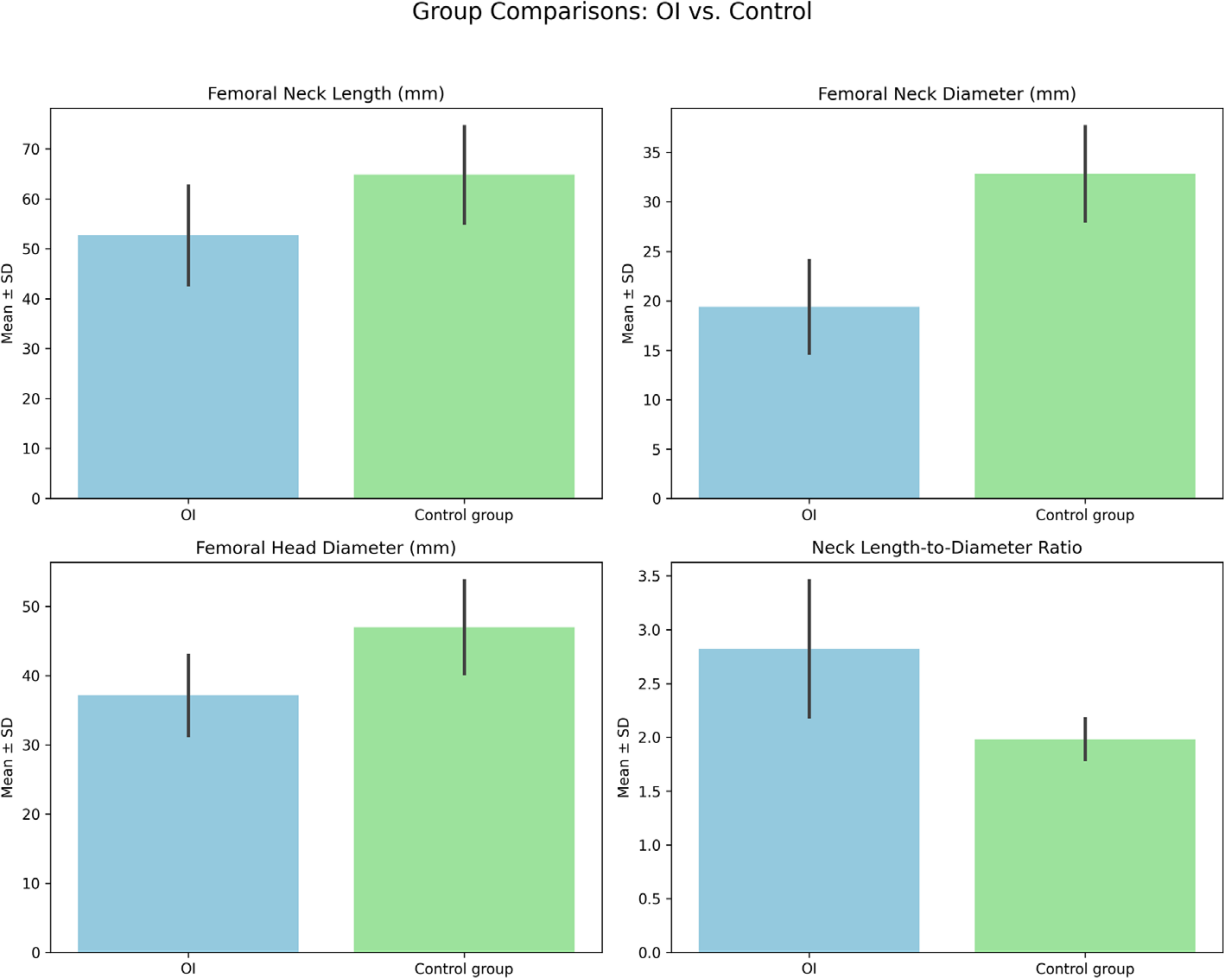
Graphs showing the distribution of femoral neck length, femoral neck diameter, femoral head diameter, and neck length-to-diameter ratio in control and OI groups.

The OI group exhibited distinct morphological differences in proximal femoral anatomy compared to controls. Femoral neck length was significantly reduced in OI patients (52.71 ± 9.85 mm) compared to controls (64.83 ± 9.62 mm, *p* < 0.001). Femoral neck diameter was also markedly smaller in the OI group (19.38 ± 4.68 mm vs. 32.83 ± 4.77 mm, *p* < 0.0001), as was the femoral head diameter (37.17 ± 5.81 mm vs. 47.00 ± 6.70 mm, *p* < 0.001) (Figure 2).

The femoral neck length-to-diameter ratio was significantly higher in OI patients (2.82 ± 0.63) than in controls (1.98 ± 0.19, *p* < 0.000001), indicating a characteristic long and slender neck morphology (Figure 2). Receiver operating characteristic (ROC) curve analysis identified an optimal cut-off value of 2.35 for this ratio to distinguish dystrophic femoral necks, yielding a sensitivity of 83%, specificity of 96%, and an area under the curve (AUC) of 0.90.

In contrast, several measured parameters did not show statistically significant differences between the groups. The femoral neck length-to-head diameter ratio was not significantly different between OI and control patients (1.44 ± 0.25 vs. 1.38 ± 0.10, *p* = 0.34). The neck-shaft angle also did not differ significantly (135.79 ± 19.15° in OI vs. 129.88 ± 4.35° in controls, *p* = 0.15), although the OI group exhibited greater variability. Similarly, the anterior Lateral Proximal Femoral Angle (aLPFA) tended to be higher in the OI group (95.04 ± 23.55° vs. 85.25 ± 6.10°), but this difference did not reach statistical significance (*p* = 0.059). Finally, no significant correlation was found between the femoral neck length-to-diameter ratio and the degree of acetabular protrusion within the OI group (*r* = 0.0012, *p* = 0.996).

**Figure 3:**
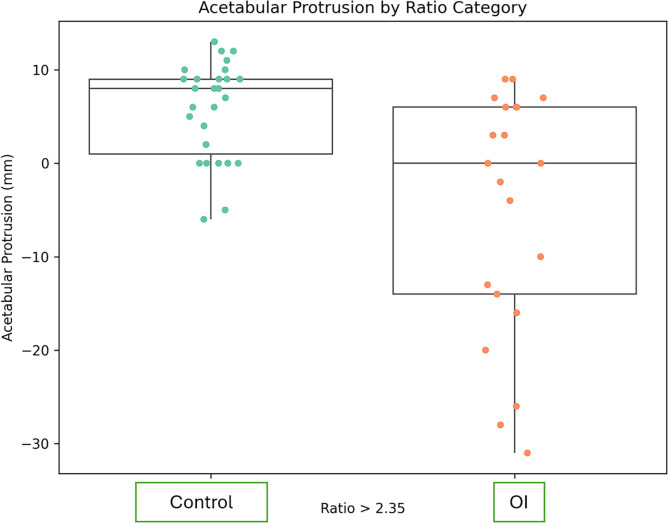
Graph showing the distribution of acetabular protrusion in patients with a femoral neck length-to-diameter ratio > 2.35 in control and OI groups.

Acetabular protrusion, measured as the distance from the acetabular floor to the ilio-ischial line, was significantly more pronounced in OI patients (–5.45 ± 12.62 mm) compared to controls (8.05 ± 3.36 mm, *p* < 0.0001), consistent with protrusio acetabuli (Table 2). Notably, some OI patients demonstrated negative values, reflecting severe medial displacement of the femoral head (Figure 3).

**Table 2:** Demographic and morphometric comparison between OI and control groups. F: Female. M: Male. aLPFA: anatomical lateral proximal femoral angle.

Parameter	OI group (*n* = 24)	Control group (*n* = 24)	*p*-value	Significant difference
Age (years)	16.1 ± 10.6	15.3 ± 9.4	0.76	No
Sex (F:M)	8:16	9:15	0.76	No
Side (Right:Left)	13:11	17:7	0.22	No
Femoral neck length (mm)	52.71 ± 9.85	64.83 ± 9.62	<0.001	Yes
Femoral neck diameter (mm)	19.38 ± 4.68	32.83 ± 4.77	<0.0001	Yes
Femoral head diameter (mm)	37.17 ± 5.81	47.00 ± 6.70	<0.001	Yes
Neck length-to-diameter ratio	2.82 ± 0.63	1.98 ± 0.19	<0.000001	Yes
Neck length-to-head diameter ratio	1.44 ± 0.25	1.38 ± 0.10	0.34	No
Neck-shaft angle (°)	135.79 ± 19.15	129.88 ± 4.35	0.15	No
aLPFA (°)	95.04 ± 23.55	85.25 ± 6.10	0.059	No (trend)
Acetabular protrusion (mm)	–5.45 ± 12.62	8.05 ± 3.36	<0.0001	Yes
Correlation (neck length-to-diameter ratio vs. protrusion)	*r* = 0.0012	–	0.996	No

### Exploratory analysis based on ROC-derived threshold

**Figure 4:**
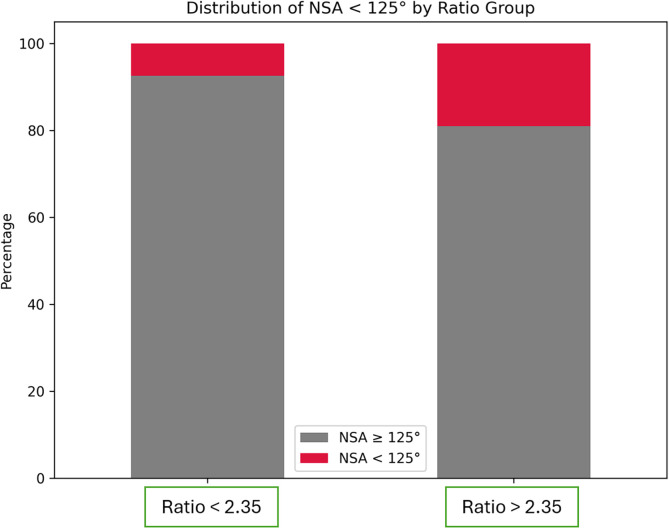
Graph showing the distribution of neck-shaft angles in OI patients with a femoral neck length-to-diameter ratio ≤ 2.35 and > 2.35. NSA: neck-shaft angle.

We explored whether the ROC-derived threshold (2.35) for the femoral neck length-to-diameter ratio was associated with other radiographic features. Among the 22 patients with a ratio >2.35, 8 had a neck-shaft angle <125°. Among the 26 patients with a ratio ≤ 2.35, only 4 had NSA < 125° (Figure 4). While this suggested a trend toward greater coronal deformity in the high-ratio group, the association did not reach statistical significance (*p* = 0.058, Fisher’s exact test) (Table 3).

**Table 3:** Exploratory subgroup analysis by ROC-derived neck length-to-diameter ratio threshold (2.35). NSA: Neck-Shaft Angle.

Parameter	Ratio > 2.35 (*n* = 22)	Ratio ≤ 2.35 (*n* = 26)	*p*-value	Significant
Patients with NSA < 125°	8/22	4/26	0.058 (Fisher’s exact)	No (trend)
Mean acetabular protrusion (mm)	–2.91 ± 12.33	4.42 ± 8.67	0.004	Yes

We also evaluated whether the high-ratio group had more severe acetabular protrusion. The mean acetabular floor position in patients with a ratio >2.35 was –2.91 ± 12.33 mm compared to 4.42 ± 8.67 mm in those with a ratio ≤ 2.35 (*p* = 0.004), indicating significantly greater protrusion in the high-ratio group.

## Discussion

In this study, we characterized the proximal femoral anatomy in patients with severe Osteogenesis Imperfecta (OI) and identified distinct morphological differences compared to age-matched controls. To our knowledge, this is the first reported radiological characterization of its kind. OI patients exhibited significantly shorter and thinner femoral necks, smaller femoral heads, and a markedly elevated femoral neck length-to-diameter ratio. This morphological profile reflects a long and slender femoral neck phenotype, which may contribute to abnormal loading patterns, compromised biomechanics, and increased fracture risk.

The significantly reduced femoral neck length and femoral neck diameter observed in the OI group were expected findings. Patients with OI typically present with reduced stature and overall skeletal underdevelopment due to impaired collagen formation and abnormal bone remodeling. These reductions in linear bone dimensions are consistent with their smaller body size relative to the general population. However, the disproportionately high femoral neck length-to-diameter ratio observed in OI patients suggests that the difference is not only due to scaling but also to abnormal geometry and altered bone modeling. This distinction highlights the importance of using shape-sensitive metrics, rather than raw size measurements, to assess structural abnormalities in this population.

The term *dystrophic femoral neck* in the context of OI refers to a structurally abnormal neck characterized by elongation, thinning, and reduced cortical density. These features likely result from a combination of intrinsic bone fragility, impaired growth, and altered mechanical loading due to reduced mobility and non-weight-bearing status. In a normal femoral neck, mechanical loading produces a well-organized trabecular architecture: compression trabeculae dominate the inferomedial region, and tension trabeculae reinforce the superolateral aspect, leading to densification particularly in the lower part of the neck [14, 15]. In contrast, dystrophic femoral necks lack these organized trabecular patterns, likely due to impaired load transmission, disuse, and bone remodeling imbalance. The femoral neck length-to-diameter ratio captures this morphological deviation quantitatively. A value greater than 2.35, as identified by our ROC analysis, reflects an imbalance between elongation and radial growth, exceeding normal anatomical proportions. This metric may thus indicate both geometric distortion and mechanical insufficiency. The elongated, thin, and low-density neck is more vulnerable to deformation and fracture, and may serve as a radiographic marker of structural instability and clinical risk.

The femoral neck length-to-diameter ratio was the most discriminative parameter. ROC curve analysis identified a threshold value of 2.35 that separated dystrophic from normal morphology with high sensitivity and specificity. In contrast, the neck length-to-head diameter ratio did not differ significantly, suggesting that this alternative metric is less sensitive to pathological elongation and narrowing of the femoral neck in OI.

We further explored whether this ROC-derived threshold could reflect broader structural consequences for the hip and pelvis. Patients with a femoral neck length-to-diameter ratio above 2.35 showed a non-significant trend toward lower neck-shaft angles (NSA < 125°), suggesting a possible link to varus alignment (*p* = 0.058). In contrast, acetabular protrusion was significantly more pronounced in the high-ratio group, indicating a potential association between femoral neck shape and pelvic remodeling. Interestingly, while the continuous correlation between the ratio and protrusion was absent, the threshold-based analysis revealed a statistically significant difference. This suggests that certain dysplastic features may manifest only beyond a structural tipping point, rather than along a linear gradient. In a series of 79 pediatric OI patients, Violas et al. found that acetabular protrusion occurred in 33% of cases, with a higher prevalence among children presenting with more severe forms of the disease [7]. Dystrophic femoral neck with acetabular protrusion could be indicators of severe forms of OI.

Importantly, these dysplastic features may impact surgical decision-making. Femoral necks that are long, thin, and less dense are prone to fractures and progressive deformation. These changes should be identified and addressed early, as delayed intervention may contribute to secondary pelvic alterations. When both acetabular protrusion and femoral neck dystrophy are present, hip mobility may be significantly reduced, potentially impairing overall function in affected patients. Early recognition of these structural abnormalities allows for timely treatment, which may help preserve joint mechanics, improve functional outcomes, and enhance quality of life [16, 17]. Most importantly, early diagnosis and management of OI – including the promotion of physical activity – may help prevent the development of dystrophic femoral necks [18–21].

This study has certain limitations. The paper did not address the interobserver and intraobserver reliability of the measurements. Furthermore, although the present series provides a comprehensive radiographic overview, a larger cohort with long-term follow-up is required to evaluate the clinical significance of the concept of a “dystrophic femoral neck.” Future studies should aim to determine the threshold at which this morphological alteration begins to influence functional outcomes or increase the risk of femoral neck fracture in patients with osteogenesis imperfecta, and will be integrated into a comprehensive algorithm treatment. Future studies with larger cohorts and 3D imaging are needed to validate the proposed cut-off and clarify the biomechanical relationships suggested here.

## Conclusion

This study identifies and characterizes a distinct dystrophic pattern of the femoral neck in patients with severe Osteogenesis Imperfecta, marked by a significantly elongated and narrowed morphology. These anatomical alterations are associated with reduced femoral head size and a high prevalence of acetabular protrusion, although no direct correlation was found between neck dystrophy and protrusion severity. ROC curve analysis demonstrated that a neck length-to-diameter ratio ≥ 2.35 reliably discriminates dystrophic femoral necks with high sensitivity and specificity. Recognition of this morphological threshold may improve radiological assessment and guide orthopedic decision-making in this fragile population.

## Data Availability

The datasets used and/or analyzed during the current study are available from the corresponding author on reasonable request.
